# Five Cryptic Species in the Amazonian Catfish *Centromochlus existimatus* Identified Based on Biogeographic Predictions and Genetic Data

**DOI:** 10.1371/journal.pone.0048800

**Published:** 2012-11-07

**Authors:** Georgina M. Cooke, Ning L. Chao, Luciano B. Beheregaray

**Affiliations:** 1 Molecular Ecology Laboratory, Department of Biological Sciences, Macquarie University, Sydney, New South Wales, Australia; 2 Bio-Amazonia Conservation International, Baltimore, Maryland, United States of America; 3 Molecular Ecology Laboratory, School of Biological Sciences, Flinders University, Adelaide, South Australia, Australia; Onderstepoort Veterinary Institute, South Africa

## Abstract

Accurately quantifying biodiversity is fundamental for both evolutionary theory and conservation strategies. DNA-based studies are exposing high cryptic diversity irrespective of taxonomic group or environmental setting, and increasing the ever-growing estimates of global biodiversity. This has severe implications for under-sampled and species-rich tropical regions, such as the Amazon Basin. We used biogeographic predictions derived from geomorphological history and contemporary hydrochemical and genetic data to examine cryptic diversity in the Amazonian driftwood catfish *Centromochlus existimatus*. Using both nuclear and mitochondrial DNA markers, five deeply divergent cryptic lineages are reported, for which at least three are sympatric in distribution. These lineages appear relatively old, with divergence times dating back to middle Miocene. Diversification events appear to be chronologically associated with the formation of the modern Amazon River system, and perhaps influenced by hydrochemical gradients between tributaries. The cause of apparent morphological stasis in the *C. existimatus* species complex is speculated within the context of hydrochemistry and non-visual mating cues and a full taxonomic revision is recommended. Our findings suggest that the diversity of Amazonian ichthyofauna is vastly underestimated and highlight the relevance of biogeographic predictions to guide sampling efforts in ecologically complex and under-studied ecosystems.

## Introduction

Species define the basic unit by which measurements of biodiversity normally are made. Although morphology-based taxonomy will continue to have a central and unrivalled position in biodiversity research [1], DNA-based studies of genetic diversity within species have exposed high cryptic diversity and cryptic species in all major terrestrial and aquatic taxonomic groups, traversing all environmental settings [1], [2], [3], [4]. Indeed, current levels of biodiversity seem to be much greater than previously suspected [5]. This has profound implications for both evolutionary theory and conservation, especially in threatened ecosystems for which biodiversity has been likely underestimated.

Tropical rainforest environments are a conservation priority, housing unparalleled concentrations of biodiversity that have become substantially threatened as a consequence of anthropogenic influence [6], [7], [8], [9], [10]. Current conservation strategies rely on guided measurements of biodiversity e.g. [5], [11]. However, in under-sampled tropical regions, such as the Amazon Basin, conservation programs rarely employ DNA-based methods of biodiversity measurement and thorough morphology-based studies. As a result, cryptic species could be insufficiently reported [3], [12], [13]. The Amazon Basin drainage system sustains the world’s richest freshwater fish fauna, with over 3000 species belonging to 60 fish families [14], [15]. While the drivers underpinning this exceptional diversity are little understood [16], genetic techniques are increasingly detecting cryptic Amazonian fish species [17], [18], [19], [20], [21], [22], [23], [24], [25], [26] indicating that species richness in this group is vastly underestimated. A similar conclusion was also recently reported for Amazonian frogs based on a comprehensive assessment of cryptic diversity [27].

Predicting spatial patterns of biodiversity is a fundamentally important component of ecological and evolutionary studies [28], [29]. Using biogeographic predictions to investigate cryptic diversity could prove useful in ecologically complex and difficult to study ecosystems, such as the Amazon Basin. Given that both historical and environmental characteristics of a region affect all stages of diversity [30], it is appropriate to consider not only the geomorphological events but also the existence of major ecological gradients when predicting spatial patterns of cryptic diversity for the extant Amazonian fish fauna. In terms of geomorphology, the formation of the Amazon Basin and river systems has been particularly influenced by the history of Andean uplift [31], [32]. Indeed, the modern west to east trans-continental Amazon River system is a product of increased sedimentation and sea level changes that occurred during the late Miocene. Briefly, these tectonic changes resulted in the overfilling of the Andean foreland basin causing the proto-Amazon River to eventually breach the Purus Arch, a ridge oriented north-south that previously divided the flow of the Amazon River [33], [34], [35]. While the trans-continental Amazon drainage system was largely formed by the late Miocene [36], [37], the full establishment of the modern Amazon River probably occurred only during the late Pliocene around 2.5 million years ago (Ma) [33].

**Figure 1:**
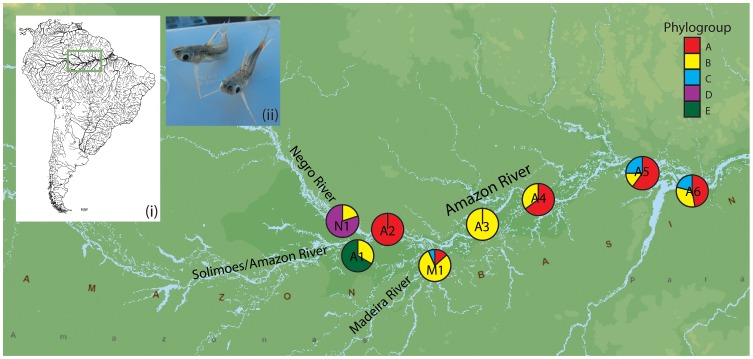
The eight sampling localities of *Centromochlus existimatus* in the Amazon Basin. The pie chart shows the proportion of individuals sampled from a site belonging to each of the five phylogroups. Inset (i) shows the location of sampling sites within South America, and inset (ii) is a photograph of *C. existimatus*.

In terms of contemporary conditions, the Amazon Basin sustains dramatic hydrochemical and ecological gradients that impose physiological constraints upon its aquatic communities [38], [39], [40], [41], [42]. For instance, based primarily on optical and sedimentary characteristics, the Amazon and Madeira Rivers (Figure 1) are known as ‘white’ water rivers, characterised by a high content of dissolved solids and a neutral pH (Table 1). The Negro River (Figure 1, Table 1) in contrast, is known as a ‘black’ water river, as its waters are acidic and transparent, stained by tannins and humic acids leached from vegetation [43]. Here we use predictions derived from these two temporal snapshots to assess cryptic diversity in a group of catfish from Amazonia.

Catfishes (order Siluriformes), represent approximately 32% of all freshwater fish diversity globally and make up a significant portion of South America’s freshwater fish fauna [14], [44]. The driftwood catfishes, or Auchenipterids, are a small to medium-sized nocturnal group of fish widely distributed throughout, and endemic to, the Neotropics [14]. Our study group is the driftwood catfish *Centromochlus existimatus* (Siluriformes: Auchenipteridae) [45], a non-migratory species found in lowland drainages of Amazonia [46]. In this study we sampled *C. existimatus* across three major rivers (the Amazon, Madeira and Negro Rivers, Figure 1) and generate genetic data to examine cryptic diversity in this group within the context of ecology and vicariant biogeographic history. From a vicariant biogeographic perspective, we expect that cryptic lineages identified in *C. existimatus* are chronologically and spatially associated with the final formation of the Amazon River system, and in particular, with the late breaking of the Miocene ridge represented by the Purus Arch. From an ecological perspective, the marked hydrochemical gradients found in the basin are predicted to influence reproductive isolation by maintaining population boundaries between contrasting selective environments, such as those found between white and black water. Using both nuclear and mitochondrial DNA makers, we unexpectedly report on five divergent lineages of *C. existimatus.* Here, we suggest that these represent cryptic species and that *C. existimatus* comprises a species complex. Consistent with the Tertiary diversification hypothesis for Neotropical faunas [31], it is found that these cryptic lineages have diversified since the middle Miocene. In light of this, we speculate a water colour based hypothesis for morphological stasis in the *C. existimatus* complex.

## Materials and Methods

*Centromochlus existimatus* was sampled from a vast area spanning over 1000 km of riverine distance of the Amazon Basin encompassing three major river systems; the Amazon, Madeira and Negro Rivers. Eight putative populations were sampled (Figure 1, Table 1) and up to 20 individuals were sampled from each population (n = 104). One specimen of *Centromochlus macracanthus*, a species adapted to rapid habitat, was sampled from São Gabriel de Cachoeira near the headwaters of the Rio Negro for outgroup inclusion in the phylogenetic analyses. Fish were caught using a beach seine net or throw net, euthanized, and muscle tissue was dissected from behind the dorsal fin and preserved in 95% ethanol. Hydrochemical variables were measured at each sampling site (Table 1). For one section of the analyses, a nuclear DNA sequence for the RAG1 gene of *Centromochlus heckelii* was obtained from GenBank (accession number DQ492562).

**Table 1:** Water ‘colour’, sampling locations and sample size of *Centromochlus existimatus* collected in the Amazon Basin.

River	Site	Latitude	Longitude	N	°C	pH	cm	O.D	O_2_%
Black water									
Negro	N1	3° 4'44.00"S	60°14'44.00"W	10	29.7	5.2	76.0	6.4	82.3
White water									
Madeira	M1	3°28'14.00"S	58°52'5.00"W	15	29.7	7.1	5.5	5.7	82.4
Amazon	A1	3°20'40.00"S	60° 7'10.00"W	3	28.8	7.2	12.3	6.7	86.3
Amazon	A2	3° 6'56.00"S	59°32'19.00"W	15	29.6	7.1	18.8	6.3	85.2
Amazon	A3	3° 4'39.00"S	58°13'13.00"W	2	28.7	7.1	18.3	4.8	84.0
Amazon	A4	2°33'7.00"S	57° 1'59.00"W	17	29.2	7.2	10.5	6.4	85.6
Amazon	A5	2°10'21.00"S	54°58'21.00"W	20	29.0	7.2	12.5	6.3	82.0
Amazon	A6	2°28'10.00"S	54°30'5.00"W	20	29.7	7.2	15	6.6	87.9

Average hydrochemical variables are also provided (Temperature, °C; pH; turbidity, cm; dissolved oxygen (mg/L); oxygen saturation O_2_ (%)).

DNA was extracted using a modified salting out method [47]. Genetic data were collected from both the nuclear and mitochondrial genomes. The mitochondrial adenosine triphosphatase subunits 6 and 8 (ATPase 6 and 8) were amplified via polymerase chain reaction (PCR) and sequenced (*n* = 104). ATPase 6 and 8 were amplified using the primers ATP8.2 (5′ AAA GCR TYR GCC TTT TAA GC) and CO3.2 (5′ GTT AGT GGT CAK GGG CTT GGR TC) [48]. Each 20 µL reaction contained 0.8 µM of each primer, 5 mM MgCl_2_, 0.4 mM each dNTP, 5× Buffer (Promega) and 1 U Taq polymerase (Promega). PCR was carried out using the following 61–53°C touchdown PCR program: 94°C for 3 min, 94°C for 30 s, 61°C for 45 s, 72°C for 1 min, 94°C for 30 s, 59°C for 45 s, 72°C for 1 min, 94°C for 30 s, 57°C for 45 s, 72°C for 1 min, 94°C for 30 s, 55°C for 45 s, 72°C for 1 min followed by 26 cycles at 94°C for 30 s, 53°C for 45 s, 72°C for 1 min, and a final extension of 72°C for 5 min.

The single copy nuclear DNA fragment of the recombination activating gene 1 (RAG1) was sequenced for a subset of the dataset (*n* = 24) using a nested PCR. For the first round PCR, RAG1 was amplified using the primers RAG1-2510F^a^ (5′ TGG CCA TCC GGG TMA ACA C) [49] and RAG1-4090R^ a^ (5′CTG AGT CCT TGT GAG CTT CCA TRA AYT T) [50]. For the second round PCR, RAG1 was amplified using the primers RAG1-2533F^b^ (5′ CTG AGC TGC AGT CAG TAC CAT AAG ATG T) and RAG14078R^ b^ (5′ TGA GCC TCC ATG AAC TTC TGA AGR TAY TT) [50]. Each 30 µL reaction contained 0.6 µM of each primer, 3 mM MgCl_2_, 0.6 mM each dNTP, 5× Buffer (Promega) and 1 U Taq polymerase (Promega). PCR was carried out using the following program: 94°C for 3 min, followed by 35 cycles at 94°C for 30 s, 55°C for 45 s, 72°C for 45 s, and a final extension of 72°C for 10 min.

**Figure 2:**
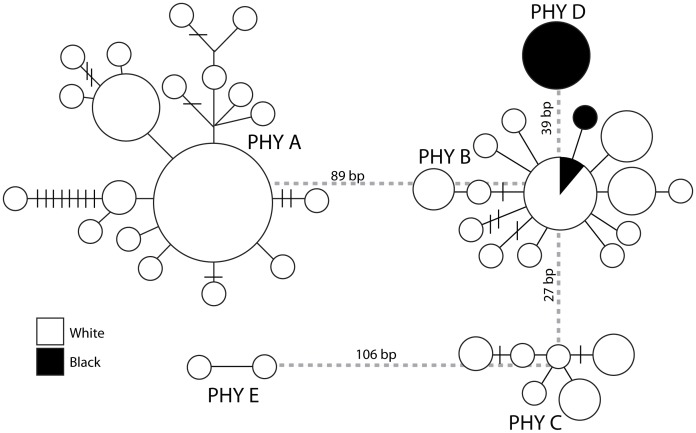
Statistical parsimony network for the *Centromochlus existimatus* species complex based on the mitochondrial haplotypes from ATPase 6 and 8. Each circle denotes a unique haplotype and the area of the circle is proportional to its frequency. The shade of the circle represents the water colour of the sample site; white or black.

Sequence data were aligned using SEQUENCHER v4.1 (Gene Codes Corporation, Ann Arbor, MI) and submitted to GenBank (accession numbers JX910142-JX910196). Genealogical relationships between all samples were investigated by constructing a haplotype network in TCS [51] using the mtDNA ATPase 6 and 8 sequence data. The TCS program estimates gene genealogies from DNA sequences using the statistical parsimony method defined by a 95% confidence interval. TCS identified five haplotype networks that did not connect at 95% statistical parsimony and herein they are referred to as “phylogroups A - E” (Figure 2). To investigate the phylogenetic relationships between these phylogroups, character-based, distance-based and model-based (Maximum Parsimony, MP; Neighbour-joining, NJ; Maximum likelihood ML and Bayesian inference, BI) methods of phylogenetic analysis were employed for both ATPase 6 and 8 and RAG1 sequence data. For each phylogeny the same five samples were used to represent each phylogroup. MP analysis was conducted using PAUP*4.0 b10 [52] using a heuristic search strategy for the most parsimonious tree with all characters considered unordered and unweighted. Bootstrap re-sampling based on 1000 replicates was used to assess the support of relationships for the majority-rule consensus tree.

For the NJ and ML phylogenetic analyses, MODELTEST 3.06 [53] was used to determine the best fit model of sequence evolution for our ATPase 6 and 8 and RAG1 sequence data. Based on the Akaike Information Criterion (AIC), General Time Reversible (+I) and the Transition model (+I) was selected respectively. The General Time Reversible model accounts for unequal base frequencies with six substitution types [54], [55], while the Transition model accounts for unequal base frequencies [53].

Corrected genetic distances based on 817 bp of ATPase 6 and 8 and 1491 bp of RAG1 were calculated using PAUP*4.0b10 [52]. NJ analyses implementing the General Time Reversible (+I) model parameters for ATPase 6 and 8 and the Transition (+I) model parameters for RAG 1 and were assessed with 1000 bootstrap replicates in PAUP*4.0b10 [52]. ML analysis was also conducted using PAUP*4.0b10 [52] employing a heuristic search strategy and appropriate model parameters as specified by MODELTEST v.3.0.6 [53].

Bayesian inference was performed in MR BAYES [56] using the best fit model of sequence evolution for both ATPase 6 and 8 and RAG1 as determined in MR MODELTEST 2 [57]. Based on the AIC, the General Time Reversible (+I) model was selected for both data sets. The Bayesian analysis was run using a Metropolis-coupled Markov Chain Monte Carlo (MCMC) algorithm from randomly generated starting trees for six million generations with sampling every 1000 generations. Both the standard deviation of splits frequencies and the potential scale reduction factor were used as a convergence diagnostic and the initial 25% of samples were discarded in the burn-in.

Cryptic speciation and speciation events were dated for the mitochondrial and nuclear data sets separately using the MCMC approach implemented in the program BEAST v1.4.6 [58]. Because there is no adequate known fossil evidence for *C.existimatus*, a molecular clock was used to estimate these dates. For ATPase 6 and 8 the molecular clock of 1.4% per million years was assumed. This clock uses divergences between geminate fish taxa across the Isthmus of Panama [59]. For RAG1 an estimated rate of 0.6 (0.04%) substitutions per million years, based on Pomacentridae fish, was employed [60]. The time to most recent common ancestor (*T*_mrca_) at each monophyletic node was estimated using the maximum likelihood ATPase and RAG1 phylogenetic trees and the concatenated maximum likelihood tree. The GTR (+I) model of sequence evolution and relaxed clock method that allows for branch specific variation, drawn from a lognormal distribution were specified [61]. Tree priors were modelled according to Yule speciation process and all other priors were set at their default values. The MCMC analyses were run for 80 million generations and sampled every 100^th^ generation with the first 10% of samples discarded as a burn-in. TRACER 1.4 [62], was used to examine results, confirm that sufficient effective sample sizes has been achieved and that stationarity had been reached. Each analysis was run twice independently to validate these results.

## Results

Of the 104 specimens identified as *C. existimatus* in the field, 42 unique mitochondrial ATPase 6 and 8 haplotypes were identified. These were distributed in five phylogroups (A -E) unconnected by 95% statistical parsimony (Figure 2). Haplotypes were characterised by 817 bps, of which 223 were variable and 187 were parsimony informative. General Time Reversible (+I) pairwise genetic differences ranged from 0.04 between phylogroups B and C to 0.18 between phylogroups A and E (Table 2).

**Table 2:** Distance matrix of pairwise genetic distances among *Centromochlus existimatus* phylogroups (A–E).

	A	A	A	A	A	A	B	B	B	B	B	C	C	C	C	C	D	D	D	D	D	E	E	CM
**A**	–	0.00	0.00	0.00	0.00	0.00	0.00	0.00	0.00	0.01	0.00	0.01	0.01	0.01	0.01	0.01	0.01	0.01	0.01	0.01	0.01	0.01	0.01	0.01
**A**	0.00	–	0.00	0.00	0.00	0.00	0.00	0.00	0.00	0.01	0.00	0.01	0.01	0.01	0.01	0.01	0.01	0.01	0.01	0.01	0.01	0.01	0.01	0.01
**A**	0.00	0.01	–	0.00	0.00	0.00	0.00	0.00	0.00	0.01	0.00	0.01	0.01	0.01	0.01	0.01	0.01	0.01	0.01	0.01	0.01	0.01	0.01	0.01
**A**	0.00	0.00	0.00	–	0.00	0.00	0.00	0.00	0.00	0.01	0.00	0.01	0.01	0.01	0.01	0.01	0.01	0.01	0.01	0.01	0.01	0.01	0.01	0.01
**A**	0.00	0.00	0.01	0.00	–	0.00	0.00	0.00	0.00	0.01	0.00	0.01	0.01	0.01	0.01	0.01	0.01	0.01	0.01	0.01	0.01	0.01	0.01	0.01
**A**	0.00	0.00	0.00	0.00	0.00	–	0.00	0.00	0.00	0.01	0.00	0.01	0.01	0.01	0.01	0.01	0.01	0.01	0.01	0.01	0.01	0.01	0.01	0.01
**B**	0.14	0.14	0.14	0.14	0.14	0.14	–	0.00	0.00	0.00	0.00	0.00	0.00	0.00	0.00	0.00	0.00	0.00	0.00	0.00	0.00	0.01	0.01	0.01
**B**	0.14	0.14	0.14	0.14	0.14	0.14	0.00	–	0.00	0.00	0.00	0.00	0.00	0.00	0.00	0.00	0.00	0.00	0.00	0.00	0.00	0.01	0.01	0.01
**B**	0.14	0.14	0.14	0.14	0.14	0.14	0.00	0.00	–	0.00	0.00	0.00	0.00	0.00	0.00	0.00	0.00	0.00	0.00	0.00	0.00	0.01	0.01	0.01
**B**	0.14	0.14	0.14	0.14	0.14	0.14	0.00	0.00	0.00	–	0.00	0.00	0.00	0.00	0.00	0.00	0.01	0.00	0.01	0.01	0.00	0.01	0.01	0.01
**B**	0.14	0.14	0.14	0.14	0.14	0.14	0.00	0.00	0.00	0.00	–	0.00	0.00	0.00	0.00	0.00	0.00	0.00	0.00	0.00	0.00	0.01	0.01	0.01
**C**	0.14	0.14	0.14	0.14	0.14	0.14	0.04	0.04	0.04	0.04	004	–	0.00	0.00	0.00	0.00	0.01	0.01	0.01	0.01	0.01	0.01	0.01	0.01
**C**	0.14	0.14	0.14	0.14	0.14	0.14	0.04	0.04	0.04	0.04	0.04	0.00	–	0.00	0.00	0.00	0.01	0.01	0.01	0.01	0.01	0.01	0.01	0.01
**C**	0.14	0.14	0.14	0.14	0.14	0.14	0.04	0.04	0.04	0.04	0.04	0.00	0.00	–	0.00	0.00	0.01	0.01	0.01	0.01	0.01	0.01	0.01	0.01
**C**	0.14	0.14	0.14	0.14	0.14	0.14	0.04	0.04	0.04	0.04	0.04	0.00	0.00	0.00	–	0.00	0.01	0.01	0.01	0.01	0.01	0.01	0.01	0.01
**C**	0.14	0.14	0.14	0.14	0.14	0.14	0.04	0.04	0.04	0.04	0.04	0.00	0.00	0.01	0.01	–	0.01	0.00	0.01	0.01	0.00	0.01	0.01	0.01
**D**	0.14	0.14	0.14	0.14	0.15	0.14	0.05	0.05	0.05	0.05	0.05	0.06	0.06	0.06	0.06	0.06	–	0.00	0.00	0.00	0.00	0.01	0.01	0.01
**D**	0.14	0.14	0.14	0.14	0.15	0.14	0.05	0.05	0.05	0.05	0.05	0.06	0.06	0.06	0.06	0.06	0.00	–	0.00	0.00	0.00	0.01	0.01	0.01
**D**	0.14	0.14	0.14	0.14	0.15	0.14	0.05	0.05	0.05	0.05	0.05	0.06	0.06	0.06	0.06	0.06	0.00	0.00	–	0.00	0.00	0.01	0.01	0.01
**D**	0.14	0.14	0.14	0.14	0.15	0.14	0.05	0.05	0.05	0.05	0.05	0.06	0.06	0.06	0.06	0.06	0.00	0.00	0.00	–	0.00	0.01	0.01	0.01
**D**	0.14	0.14	0.14	0.14	0.15	0.14	0.05	0.05	0.05	0.05	0.05	0.06	0.06	0.06	0.06	0.06	0.00	0.00	0.00	0.00	–	0.01	0.01	0.01
**E**	0.17	0.17	0.17	0.17	0.17	0.17	0.16	0.16	0.16	0.16	0.16	0.16	0.16	0.16	0.16	0.15	0.17	0.17	0.17	0.17	0.17	–	0.00	0.02
**E**	0.17	0.17	0.17	0.17	0.18	0.17	0.17	0.16	0.16	0.16	0.16	0.16	0.16	0.16	0.16	0.15	0.18	0.18	0.18	0.18	0.18	0.00	–	0.02
**CM**	0.16	0.16	0.16	0.16	0.16	0.16	0.14	0.13	0.13	0.13	0.13	0.14	0.14	0.14	0.14	0.14	0.15	0.15	0.15	0.15	0.15	0.19	0.19	–

The bottom diagonal is General Time reversible model (+I) corrected genetic distance based on 817 bp of the mtDNA ATPase 6 and 8 genes. The top diagonal is Transition model (+I) corrected genetic distances based on 1491 bp of the nuDNA RAG1 gene. ‘CM’ denotes *C. macracantus.*

Based on the genealogical relationships of haplotypes (Figure 2) and their geographic distribution as phylogroups (Figure 1), it appears that phylogroups A, B and C are largely sympatric along the Amazon and Madeira Rivers. These two rivers are classified as ‘white water’ rivers [43], and measured similar hydrochemical data having high levels of sediment and a neutral pH (Table 1). In contrast, phylogroup D was found exclusively in the Negro River (Figure 1, 2). The Negro River (pH∼5) is more acidic than the Amazon or Madeira Rivers (pH∼7) and although tannin stained, turbidity in the Negro River (∼76 cm) is lower than the Madeira or Amazon Rivers (5–29 cm) (Table 1). Phylogroups A and C were not sampled west of the confluence of the Negro and Amazon Rivers, while phylogroup B was sampled at every location, except A2. Sample sizes in A1 and A3 were too small to be representative and as a result the population compositions here should be interpreted with caution. Furthermore, phylogroup E (n = 2) was only found west of the Negro River in A1.

**Figure 3:**
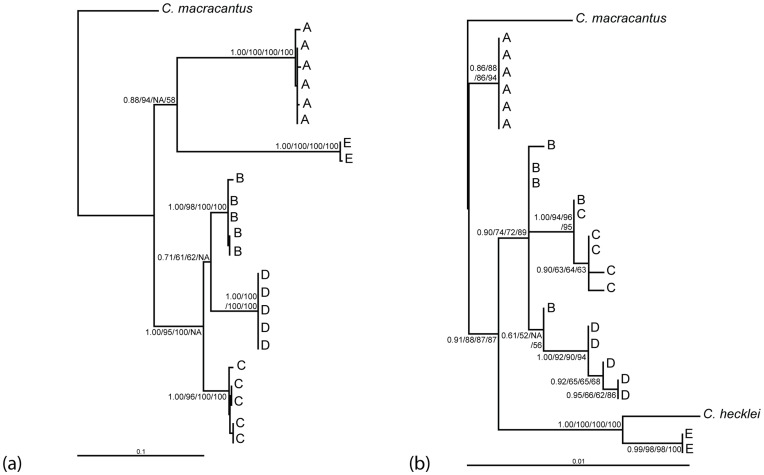
Maximum likelihood trees showing phylogenetic relationships for the *Centromochlus existimatus* species complex. Phylogenetic relationships are inferred from sequences of the mitochondrial ATPase 6 and 8 genes (a) and the nuclear RAG1 gene (b). Codes A–D correspond to the five different phylogroups, as in Figure 1. Numbers below branches are the posterior probability and bootstrap support values base on BI/ML/MP/NJ analysis respectively.

Each method of phylogenetic analysis for the mtDNA ATPase 6 and 8 resulted in largely concordant tree topologies supporting the monophyly of each phylogroup. However, for MP analysis, phylogroup E appears basal to B, C, and D, but with poor bootstrap support ( = 63). Furthermore, for NJ analysis, phylogroup D appears basal to B and C. For this reason we present only the ML phylogram with bootstrap values and posterior probabilities relevant for each method (Figure 3, a). In this tree, phylogroups B, C and D form a closely related monophyletic lineage, while phylogroups A and E form an additional clade, in which A and E are divergent from each other.

The nuclear RAG1 sequence data were characterised by 1491 bps of which 40 were variable and 28 were parsimony informative. Transition model (+I) pairwise distances ranged from 0.0007 between phylogroups B and C to 0.01 between phylogroups D and E (Table 2: all genetic distances rounded to two decimal places). For the RAG1 data set, each method of phylogenetic analysis resulted in consistent topologies (Figure 3, b). In this tree however, the monophyly of phylogroups B, C and D is not supported. Furthermore, unlike the mtDNA phylogenetic analysis, phylogroup E appears basal to B, C and D, while phylogroup A appears as the most basal phylogroup. Additionally *C. heckelii* forms a monophyletic clade with phylogroup E with good statistical support. Nonetheless, considerable genetic distances were reported between these lineages for this slowly evolving gene (Transition model (+I) pairwise distances = 0.013).

**Figure 4:**
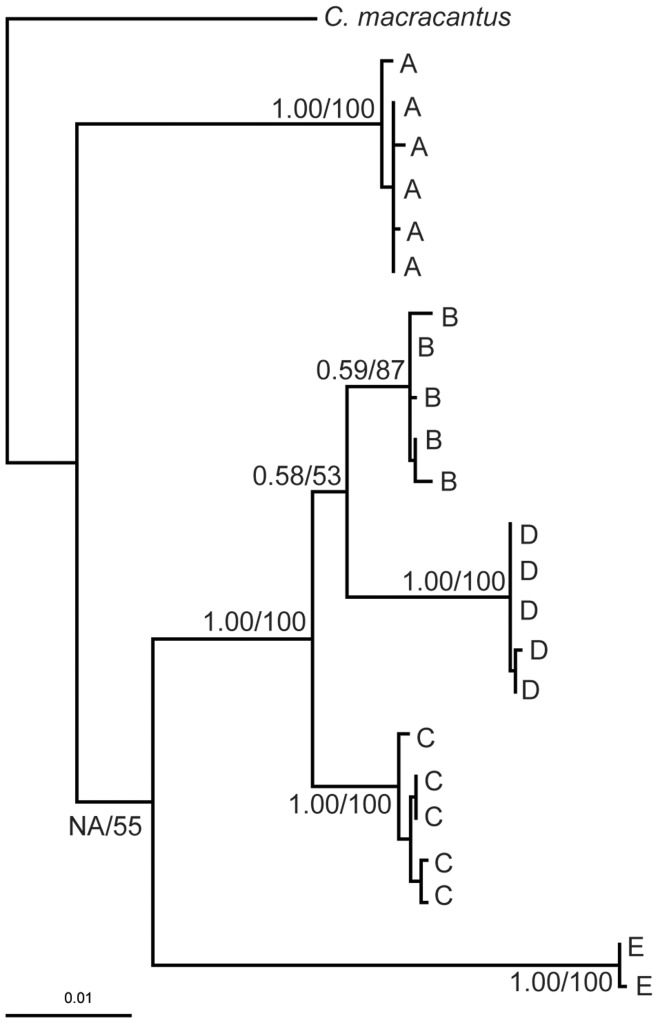
Maximum likelihood tree showing the phylogenetic relationships for the *Centromochlus existimatus* species complex. Phylogenetic relationships are inferred from the concatenated data set including mitochondrial ATPase 6 and 8 genes and the nuclear RAG1 gene. Numbers below branches are the posterior probabilities based on Bayesian inference and bootstrap support values based on Maximum likelihood analysis.

The monophyly of clades A – E were supported with the concatenated mtDNA and nuDNA phylogenetic analyses (Figure 4). BI inference had a similar topology to the mtDNA ATPase phylogenetic analysis however the model did not achieve convergence. Because of this, the results from the concatenated ML tree are presented in which model parameters were not specified for each gene. This tree is consistent with the RAG1 phylogenetic analysis in which phylogroup A, which was the most frequently sampled phylogroup (Figure 2), appears basal to all other phylogroups, but with poor bootstrap support. The monophyly of each phylogroup has strong bootstrap support, however the relationship between phylogroups B and D is not strongly supported.

**Figure 5:**
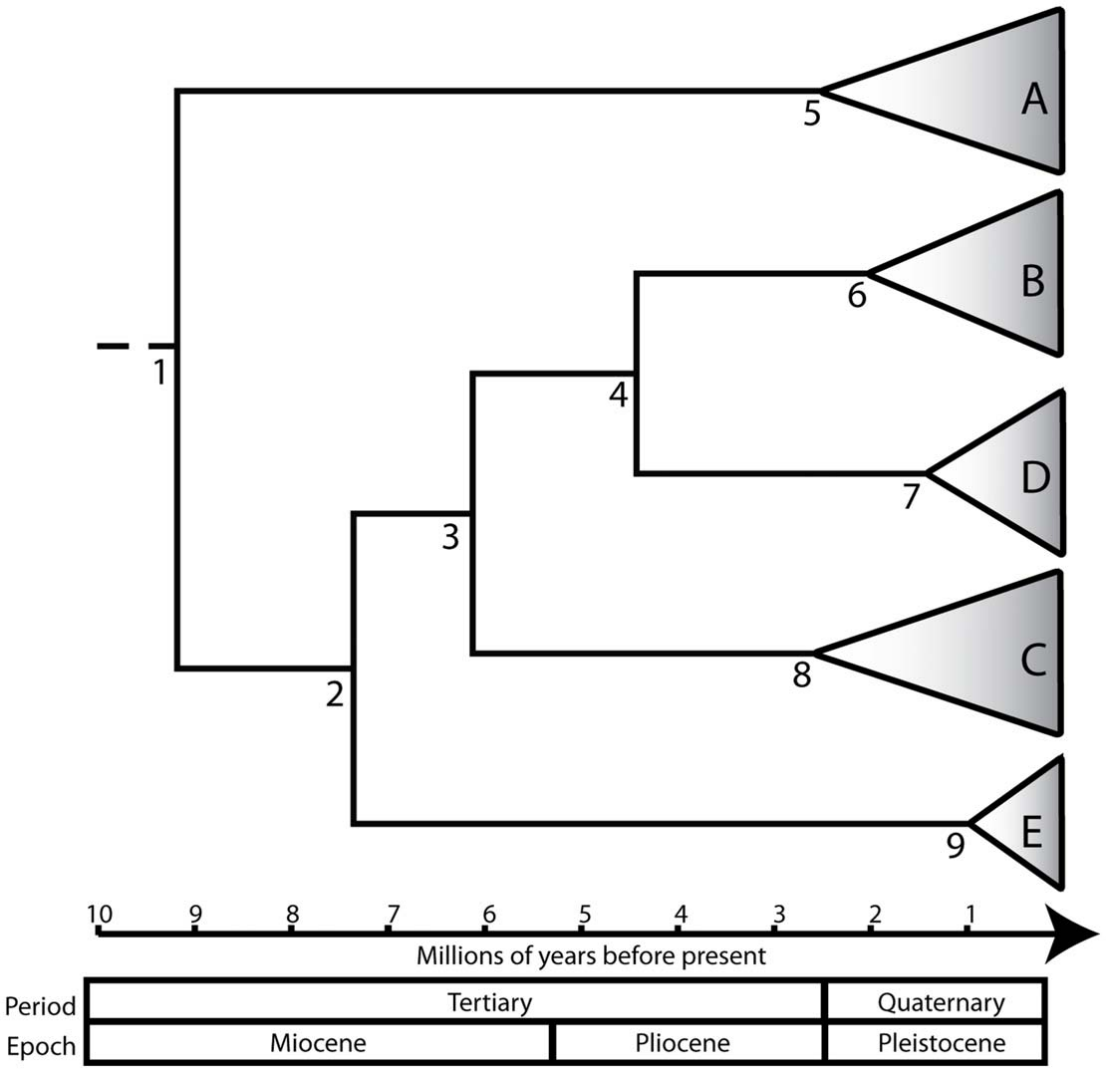
Chronogram showing divergence date estimates for the *Centromochlus existimatus* species complex in the Amazon Basin. The time to most recent common ancestor (T*_mrca_*) estimates for nodes 1–9 and their 95% lower and upper highest probability densities are provided in Table 3.

Time to most recent common ancestor was estimated for each monophyletic node using the ATPase 6 and 8, RAG 1 and concatenated ML phylogenetic trees (Figure 5, Table 3). Based on these estimates, diversification probably began within the *C. existimatus* species complex during the middle to late Miocene. For the nodes 1, 2 and 3, estimates based on the RAG data were generally higher, with wider confidence intervals than those based on the ATPase 6 and 8 or concatenated data. Nonetheless, the analyses taken together support splits between phylogroups B, C and D during the end of the Miocene (∼6 Ma), while phylogroups B and D likely arose more recently during the Pliocene (∼4.5 Ma).

**Table 3:** Time to most recent common ancestor (*T*_mrca_) statistics and 95% lower and upper highest probability densities as calculated in BEAST v.4.1.8 for the mitochondrial genes ATPase 6 and 8, the nuclear gene RAG1 and the concatenated data set.

Node number	*T*_mca_ (Ma)			95%HPD lower (Ma)			95% HPD higher (Ma)		
	ATPase 6, 8	RAG	Concatenated	ATPase 6, 8	RAG	Concatenated	ATPase 6, 8	RAG	Concatenated
1	11.185	15.205	9.155	5.370	7.557	5.156	18.209	23.920	15.133
2	8.633	12.028	7.537	4.204	6.079	4.450	14.044	19.200	12.643
3	4.441	9.487	6.125	1.764	4.619	2.838	7.640	15.289	10.606
4	2.805	NA	4.479	0.932	NA	1.608	5.045	NA	8.2180
5	1.453	NA	2.682	0.195	NA	0.377	3.843	NA	6.6141
6	0.75	NA	2.024	0.108	NA	0.395	1.786	NA	4.4650
7	1.184	NA	1.514	0.188	NA	0.128	2.810	NA	3.8272
8	0.488	NA	2.682	0.002	NA	0.559	1.386	NA	6.0388
9	0.476	NA	1.101	0	NA	0	1.529	NA	2.9505

## Discussion

Here, using molecular phylogenetic evidence, a minimum of five deeply divergent cryptic species of driftwood catfish *Centromochlus existimatus* are reported in the Amazon Basin. Results from the concatenated nuclear and mitochondrial DNA dataset support the monophyly of five *C. existimatus* phylogroups with divergences dating back from the middle Miocene to the Pliocene – this includes lineages that appear reproductively isolated in sympatry (Figure 1). Thus, these results satisfy operational criteria for application of the phylogenetic species concept [63], [64], [65], and the biological species concept [66], [67]. These findings indicate that shared ancestral polymorphisms have been lost either as a product of stochasticity or divergent natural selection, while the absence of shared genes is indicative of either strict reproductive isolation or ongoing selection against gene exchange between phylogroups. Within the slowly evolving nuclear DNA dataset however, there appears to be incomplete lineage sorting and/or hybridization among three recently derived phylogroups (B, C and D: Figure 3, b). This fact does not necessarily imply that phylogroups B, C and D are not discrete species; rather, it may be likely that they are simply incipient species in which some hybridization may have occurred and shared ancestral polymorphisms remain within their genomes. Indeed, it has been found that nearly one quarter of all animal species are not monophyletic based on the faster evolving mtDNA genomes [68]. Considering the slower rate of nuclear to mitochondrial evolution, and the smaller effective population size of mitochondrial DNA [30], [69], the monophyly of each phylogroup in our combined mtDNA and nuDNA phylogenetic analyses and their largely sympatric distributions present a strong case for five cryptic *C. existimatus* species.

A popular assumption about cryptic species is that they are the product of recent speciation events in which morphological traits and other diagnostic characters have not had the time to evolve [1]. While this may be the case in some taxa e.g. [70], this assumption has been challenged when ancient divergences are observed between cryptic taxa e.g. [13], [22], [24], [71], [72], [73]. We show that cryptic lineages of *C. existimatus* have probably diverged since the middle Miocene, with the most recent split occurring between phylogroups B and D during the early Pliocene (Figure 5, Table 3). These speciation events took place well before the climatic oscillations of the Quaternary which were thought to have initiated rapid speciation across the world, particularly in Europe and North America [74]. In the Neotropics however, it has recently been shown through molecular dating (including for invertebrates, amphibians, fish, reptiles, birds, mammals, and plants) that most lineage splits date to the late Eocene and early Oligocene [31]. Rather than climatic oscillations of the Quaternary, Neotropical speciation has largely been attributed to paleogeographic changes associated with Andean orogeny, marine transgressions into South America and the closure of the Isthmus of Panama during the Tertiary [31], [37], [75].

In terms of paleogeography within South America, the period from the middle Miocene to early Pliocene was especially relevant to the Amazonian fluvial system. In particular, it was during this time that the Amazon River and its tributaries were establishing their west-to-easterly transcontinental flow. This development opened up new available aquatic habitats and fused the previously isolated western and eastern biota [36], [37]. While divergence date estimates calculated using non-specific molecular clocks must be interpreted with caution [76] our T*_mrca_* estimates indicate that the *C. existimatus* species complex may have begun radiating around the time of formation of the modern Amazon River (8–4 Ma) (Figure 5, Table 3). The modern Amazon River formed as a compound response following the overfilling of the Andean foreland basin and the breaching of the sedimentary basin known as the Madre de Dios formation situated just west to where the Negro River outflows into the Amazon River [33], [36], [37], [77]. Interestingly, the most basal phylogroups in our concatenated phylogeny are A and E, these phylogroups were sampled in the white water habitats of the Amazon and Madeira Rivers, but not in the black waters of the Negro River. Moreover, the most recently derived phylogroups B and D were both sampled in the black waters of the Negro River, with D apparently found exclusively in that system. This pattern may suggest that the ancestral ecotype of *C. existimatus* existed in the westerly turbid Andean derived waters and colonized the Amazon River system in an easterly direction following the overfilling of the Andean foreland basin. Subsequent to this colonization, reduced competition in the black waters of the Negro River habitat may have facilitated its recent adaptation and speciation. Thus, in light of the age and distribution of the *C. existimatus* species complex throughout the Amazon, Madeira and Negro Rivers, it is likely that the paleogeographic events involved in the genesis of new habitat within the Amazon basin may have encouraged diversification. Considering the ancient divergences between cryptic lineages of *C. existimatus*, there are several reasons that may account for its apparent morphological stasis. Firstly, species may become less morphologically diverse in scenarios of high ecological opportunity and lineage diversity. It has been shown that the rate of morphological evolution slows down as time proceeds and, further, is negatively correlated with species diversity [78]. In this way, cryptic species should not be unexpected in Amazonia since it is not only species rich, but it also has an ancient history of diversification [31]. Other contributing factors for morphological stasis may include non-visual reproductive signals, and/or selective pressures that promote morphological stasis among cryptic lineages [1]. While selection for morphological stasis appears more likely under extreme environmental conditions [79], [80], it is probable that *C. existimatus* communicate reproductive signals, at least in part, via non-visual means. Indeed, Auchenipterids like *C. existimatus* are nocturnal, retreating to deeper waters, often in crevices or submerged logs during the day [14]. They also inhabit the exceptionally turbid ‘white’ waters of the Madeira and Amazon River (turbidity 5–20 cm), and the translucent yet tannin stained ‘black’ waters of the Negro River (turbidity ∼75 cm) (Table I). Within these aquatic environments, the communication of visually based mating signals would be challenging. Yet despite this, Auchenipterids, are thought to undergo insemination in which females may then carry unfertilized mature eggs and sperm packets inside their reproductive organs for extended periods of time, prior to triggering fertilization and egg discharge [14]. This sort of mating system would require sophisticated mate recognition strategies that are likely to include non-visual signals in the optically complex Amazonian aquatic environments. Furthermore, these non-visual signals would be particularly important for mate selection where cryptic species have sympatric distributions (e.g. phylogroups A, B and C, Figure 1).

Considering the extreme contrast in optical characteristics of Amazonian waters between rivers systems (e.g. black vs. white water), reproductive adaptations may also differ between water colours. Indeed, based on the distribution of cryptic species, phylogroup D was not sampled in either ‘white’ water river, while phylogroups A and C were not sampled in the ‘black’ waters of the Rio Negro. Because the marked hydrochemical discontinuities between the Negro and Amazon Rivers are known to impose physiological constraints upon aquatic communities [38], [39], [41], [42], this result may be a preliminary signature of ecologically based divergent natural selection [81], [82]. In this case, sexual isolation may have evolved as a consequence of the ecologically driven adaptive divergence of mating cues between black and white waters [83], [84]. This tentative hypothesis of ecological speciation in *C. existimatus* should be assessed further using information from phenotypic data (including behavioral and/or physical isolation by sperm shape [85]) and from highly resolving multilocus datasets for populations sampled across the hydrochemical gradients found in Amazonia. In fact, this hypothesis is corroborated by our ongoing projects on Amazonian fish speciation that support ecological divergences and speciation driven by water colour. The latter includes studies based on multilocus datasets for three fish species (an electric fish, a puffer, and a characin) [86], [87], [88] sampled from the very same sites as *C. existimatus* and from a phylogeographic survey of a marine invader, a croaker [26].

Here we present the first molecular DNA study on *C. existimatus* and provide strong evidence for up to five cryptic species within the Amazon Basin. Our findings suggest that the diversity of Amazonian ichthyofauna is vastly underestimated and highlight the relevance of biogeographic predictions to guide sampling efforts in ecologically complex and under-studied ecosystems. This study also suggests the need for a taxonomic revision in this group and provides a baseline for future research on the evolutionary mechanisms behind cryptic speciation within the *C. existimatus* species complex involving paleogeography and non-visual mating cues associated with hydrochemistry. Considering that non-visual reproductive signals may drive cryptic speciation, we believe that cryptic fish species may be relatively common in the turbid ‘white’ and tannin stained ‘black’ Amazonian aquatic environment [26], [86], [87], [88]. This has important implications in the conservation of biodiversity in Amazonia, since the quantification of biodiversity is fundamental to accurately explaining it and ultimately conserving it [1].
